# Application of machine learning models in pharmaceutical engineering for prediction of pharmaceuticals solubility in supercritical solvent: study on phenytoin solubility

**DOI:** 10.3389/fchem.2026.1775080

**Published:** 2026-03-13

**Authors:** Shengnan Yu, Yang Chen, Weidong Qiang

**Affiliations:** School of Medicine, Huanghuai University, Zhumadian, China

**Keywords:** drug solubility, gamma regression, K-nearest neighbors, polynomial regression, supercritical processing

## Abstract

This research investigates the predictive performance of ensemble learning models, specifically Bagging, when combined with weak models including Polynomial Regression (PR), K-Nearest Neighbors (KNN), and Gamma Regression (GR) to estimate drug solubility in supercritical carbon dioxide as the solvent. The models were trained and optimized using the Bat Algorithm (BA). The objective was to accurately predict two important properties: CO_2_ density and the solubility of phenytoin in it. The bagging technique was applied to combine the predictions of multiple weak models, enhancing overall performance. The results demonstrated remarkable predictive capabilities of the Bagging model with Polynomial Regression (BAG + PR) for both CO_2_ density and drug solubility. It achieved a high *R* ^2^ score of 0.9949 for CO_2_ density and 0.97833 for solubility. The BAG + PR model also exhibited the lowest Root Mean Square Error (RMSE), indicating superior accuracy in predictions. Moreover, it exhibited the lowest Average Absolute Relative Deviation (AARD%) and Maximum Error, further validating its effectiveness in accurately capturing the relationships among the variables. Comparatively, the BAG + KNN and BAG + GR models also performed well but fell short of the BAG + PR model. While they showed respectable *R* ^2^ scores, their RMSE values were higher, suggesting larger prediction errors. The AARD% and Maximum Error metrics were also higher for these models, indicating less precise and more variable predictions.

## 1 Introduction

Improving solubility of pharmaceuticals is a key challenge in this industry because of the low water solubility of some medicines which make them difficult to dissolve in the body. As such, more drug dosage must be taken to reach the desired effects in the body of patients. Consequently, more drug dosage will result in more side effects. Thus, several methods have been employed recently to enhance the solubility of medicines in water such as nanomedicine which possess higher surface area and surface energy. Also, amorphization is another method which transforms materials from crystalline to amorphous state which possess higher solubility (G et al., 2018; Hempel et al., 2021; Qiang et al., 2024).

For preparation of medicines with greater solubility, one needs to correlate the solubility values to process parameters so that the solubility could be predicted over a wide range of parameters such as pressure and temperature. In continuous mode of operation, the flow rate is also of great importance. Solubility of medicines in solvents can be correlated via different techniques such as thermodynamics and data-driven models (Ghazwani and Yasmin Begum, 2023; Khorsandi et al., 2022; Liu et al., 2022a; Obek et al., 2024). For preparation of nanoparticles of drugs, supercritical solvents can be employed as these are green solvent and also compressible. In fact, the solubility of drugs in supercritical solvents can be tuned by variation of pressure and temperature (Amani et al., 2025; Ghazwani and Yasmin Begum, 2023).

Machine learning (ML) is recently revolutionizing various industries and domains by enabling automated learning and decision-making processes from data. With the increasing availability of large datasets and advancements in computational power, ML algorithms have gained significant popularity and effectiveness (Mitchell, 2006). Recently, ML techniques have been applied in the pharmaceutical domain to model and predict the solubility of drugs in supercritical carbon dioxide as a solvent (Alsaab and Althobaiti, 2025a; Kostyrin et al., 2022; Li et al., 2023; Liu et al., 2022b).

Various algorithms of ML can be utilized for solubility correlation among which, ensemble learning models and regressive methods are of great accuracy for fitting the solubility dataset. The Gamma regression method is a specialized form of regression analysis that is intended to model continuous variables that are non-negative and follow a distribution that is gamma in nature. This phenomenon is especially advantageous in cases where the dependent variable exhibits a non-symmetrical distribution, and its dispersion is linked to its average value. Through the incorporation of the distinctive attributes of the gamma distribution, this regression model offers a resilient structure for the examination and projection of outcomes that are not negative in nature.

Polynomial regression constitutes a flexible modeling approach for representing nonlinear associations between explanatory variables and an outcome of interest. Through the inclusion of higher-order terms, the method accommodates curvature and more intricate structural patterns in the data, thereby improving predictive performance when linear assumptions are insufficient. A complete treatment involves specifying the functional form, estimating parameters, assessing model adequacy, and interpreting fitted coefficients. Together, these elements support a rigorous analysis of variable relationships and yield insights that inform sound, data-driven decisions across a range of applications.

KNN model is a powerful non-parametric regression technique that relies on the concept of similarity to make predictions. It provides flexibility, adaptability, and intuitive reasoning, making it suitable for a wide range of regression problems. The model formulation, choice of k, distance metric selection, evaluation methods, and applications contribute to a comprehensive understanding of KNN regression as a valuable tool for predictive insight.

In this research we employed bagging ensemble model and Polynomial Regression (PR), K-Nearest Neighbors (KNN), and Gamma Regression (GR) as base models and Bat Algorithm as hyper-parameter optimizer to make models on CO_2_ density and the solubility of phenytoin in the solvent. For the first time, a combination of machine learning models and optimization techniques has been developed to relate the solubility of phenytoin in supercritical CO_2_, as well as solvent density, to variations in pressure and temperature.

## 2 Drug dataset

**FIGURE 1:**
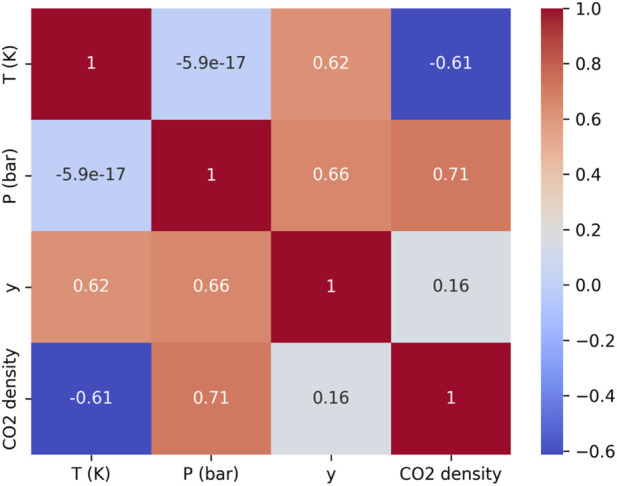
The heatmap of correlations between variables.

In this study, the goal is to develop models for predicting the solubility of phenytoin (y) and the density of carbon dioxide (CO_2_ density) versus temperature (T) in Kelvin and pressure (P) in bar. The dataset consists of 32 observations of these variables as extracted from (Notej et al., 2023). The complete dataset is presented in Table 1, while Figure 1 illustrates a heatmap showing the correlations between the variables. Data are the experimental measurements of drug solubility in the solvent at different conditions such as T and P (Alsaab and Althobaiti, 2025a; Ghazwani and Yasmin Begum, 2023).

**TABLE 1:** Experimental data for modeling (Notej et al., 2023).

T (K)	P (bar)	y	CO_2_ density
313	95	0.75	576.5599
313	110	1.06	682.684
313	130	1.24	743.5779
313	145	1.51	772.9667
313	160	1.79	795.9429
313	180	2.12	820.6486
313	200	2.46	841.0035
313	250	3.01	880.7093
318	95	0.68	424.3987
318	110	1.12	600.9256
318	130	1.64	693.3373
318	145	2.25	732.1035
318	160	2.76	760.79
318	180	3.08	790.1662
318	200	3.6	813.7248
318	250	5.05	858.2483
333	95	1.1	262.686
333	110	1.47	362.7499
333	130	2.36	507.4301
333	145	3.83	584.1816
333	160	5.14	637.4255
333	180	7.16	687.4706
333	200	9.02	724.1375
333	250	12.03	787.3101
345	95	2.15	222.2849
345	110	3.04	286.4008
345	130	4.09	390.1502
345	145	6.27	467.5024
345	160	8.18	532.2831
345	180	10.61	597.9683
345	200	12.27	646.4997
345	250	15.7	727.3968

## 3 Methodology

### 3.1 BAT algorithm

The Bat Algorithm is a population-based metaheuristic algorithm that draws inspiration from the natural world, specifically the echolocation abilities of bats for navigation. The algorithm is composed of a collection of bats, where each bat is characterized by a frequency and a position within the search space. The search strategy is guided by the bats’ movement dynamics, specifically their velocity and frequency of flight. The aforementioned parameters are subject to revision during each iteration, whereby the current positions of the bats and the positions of the most optimal bats in the population are taken into consideration (Taha and Tang, 2013; Thajudeen et al., 2025; Yang, 2013).

The equation utilized for updating the velocity and position of each bat is presented below (Fister, 2014; Thajudeen et al., 2025; Yang and Gandomi, 2012):

$$\vec{v_{i}}\left(t+1\right)=\vec{v_{i}}\left(t\right)+\vec{F_{i}}\left(t\right)\cdot \left(\vec{X_{\text{best}}}-\vec{X_{i}}\left(t\right)\right)$$

$$\vec{X_{i}}\left(t+1\right)=\vec{v_{i}}\left(t+1\right)+\vec{X_{i}}\left(t\right)$$

The term “velocity” ( $\vec{v_{i}}\left(t\right)$ ) refers to the rate at which the *i*th bat is moving at a particular iteration (t). The vector $\vec{F_{i}}\left(t\right)$ represents a random vector that influences the bat’s behavior. The random vector helps control the loudness and pulse rate of the bat’s echolocation signal, which in turn affects its ability to exploit and explore the search space available for optimization (Thajudeen et al., 2025).

### 3.2 Bagging regression

Bagging regression is an ensemble technique that aggregates the predictions of several base regression models to generate a more reliable and precise overall prediction. By utilizing the diversity of individual models, it reduces variance and enhances the stability of predictions (Breiman, 1996; Li et al., 2024).

This ensemble method involves several key steps. Initially, the training dataset is randomly partitioned into several subsets, called bags, using bootstrap sampling. Each bag represents a different subset of the original data, introducing variability into the training process.

Next, independent base regression models are trained on each bag. These base models can be any suitable regression algorithm, such as decision trees, linear regression, or support vector regression. Each base model is trained on a distinct subset of the data, promoting variability and diversity within the ensemble. Once the base models are trained, they individually produce predictions for the test dataset. The overall prediction is then derived by combining these individual outputs. In regression problems, this is commonly achieved through simple averaging, though alternative aggregation strategies (Li et al., 2024).

**FIGURE 2:**
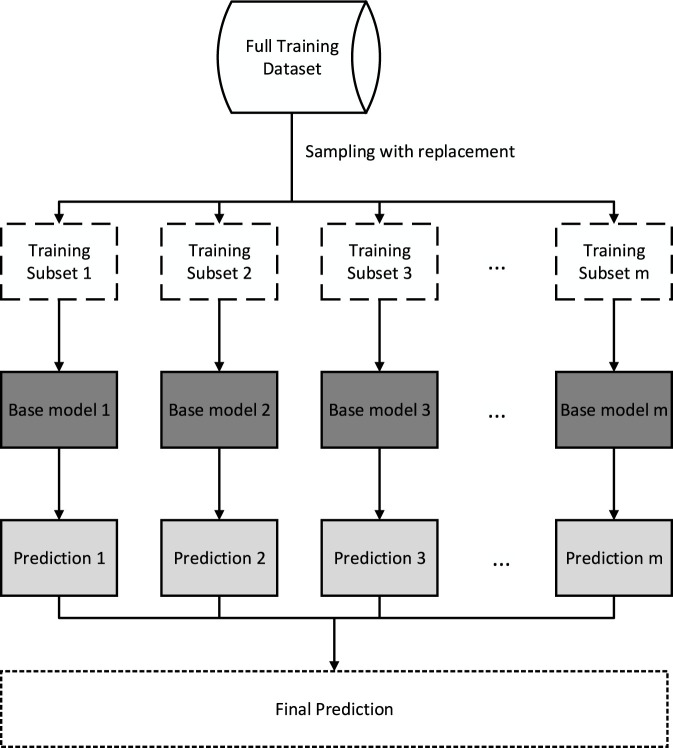
Schematic workflow of Bagging.

Bagging regression provides adaptability in choosing base learners, allowing the use of variant regression algorithms tailored to the nature of the dataset and the requirements of the task. It provides an effective solution for both nonlinear and linear regressions, adapting to the complexity of the problem at hand (Erdal and Karahanoğlu, 2016). Figure 2 illustrates the schematic flowchart of bagging ensemble model (Li et al., 2024).

### 3.3 Polynomial regression (PR)

This model enables the modelling of associations between predictors and the response variable through the utilization of polynomial functions. Polynomial regression builds on traditional linear regression by incorporating higher-degree terms, allowing it to model nonlinear trends within the dataset. This approach offers flexibility in capturing complex relationships, making it a valuable tool for revealing underlying patterns in the data (James, 2013; Ostertagová, 2012).

In PR, the relationship between the target variable *Y* and the features $X_{1},\ldots ,X_{p}$ is indicated using polynomial terms of different degrees. The general form of a polynomial regression equation is (Hastie et al., 2015):

$$Y=\beta_{0}+\beta_{1}X_{1}+\ldots +\beta_{p}X_{p}+\beta_{11}X_{1}^{2}+\beta_{12}X_{1}X_{2}+\ldots +\beta_{pp}X_{p}^{2}+$$

where *Y* stands for the target variable, $X_{i}$ are the predictors, and $\beta_{i}$ coefficients represent the weights assigned to each predictor and its polynomial terms.

### 3.4 Gamma regression (GR)

GR model assumes that the target variable *Y* follows a gamma distribution characterized by a shape parameter *k* and a scale parameter θ . The relationship between the mean (*μ*) and the predictors (*X*) is modeled using a log link function (Alkhammash, 2024). The model can be expressed as (Alkhammash, 2024; McCullagh and Nelder, 1989; Yee, 2015):

$$g\left(\mu \right)=\beta_{0}+\beta_{1}X_{1}+\ldots +\beta_{p}X_{p}$$

where *g()* stands for the log link function and $\beta_{0},\beta_{1},\ldots ,\beta_{p}$ indicate the regression coefficients associated with the features $X_{1},\ldots ,X_{p}$ .

Maximum likelihood estimation (MLE) is a widely adopted technique for determining the parameters of a gamma regression model. This method aims to identify the coefficient values that optimize the probability of observing the sample (Ghazwani et al., 2023; Schworer and Hovey, 2004). In gamma regression, the coefficients are interpreted on a logarithmic scale due to the log link function. Each coefficient $\beta_{i}$ stands for the proportional change in the expected response for a one-unit increase in the corresponding predictor $X_{i}$ , considering all other variables remain constant. The coefficient’s sign reveals whether the predictor has a positive or negative effect on the response variable (Müller and Welsh, 2009).

### 3.5 K-nearest neighbors (KNN)

KNN regression is a ML technique designed to predict continuous outcomes by leveraging the values of the nearest data points in the feature space. The algorithm is characterized as non-parametric and lazy, as it does not make any assumptions regarding the data distribution and solely relies on the training set when it is actively employed. The KNN operates under the assumption that data points that are similar in nature are likely to exhibit similar outcomes (Bishop, 2006; Trevor et al., 2009).

The KNN algorithm utilizes the identification of the K closest samples in the training set to a given new data point, enabling the prediction of the output value for the latter. The selection of a hyperparameter, specifically the value of K, is a prerequisite for the training of an algorithm. The algorithm begins by measuring the distances between the new data point and all training points, then selects the *K* nearest neighbors. The prediction is computed as the average of the outputs of these *K* closest points (Kramer et al., 2013).

KNN regression can utilize a range of distance measures, including Manhattan distance, Euclidean distance, and cosine similarity, to determine the proximity between data points. The Euclidean distance is the prevailing metric employed in KNN regression. Its computation involves the following steps (Taunk, 2019):

$$d\left(x_{i},x_{j}\right)=\sqrt{\sum_{k=1}^{p}{\left(x_{ik}-x_{jk}\right)}^{2}}$$

where *x*_*i*_ and *x*_*j*_ stand for two data points with *p* features.

The predicted output value for the new data point is (Taunk, 2019):

$$\hat{y}=\frac{1}{K}\sum_{i=1}^{K}y_{i}$$

where *y*_*i*_ denotes the output value of the *i*th closest data point to the new data point.

## 4 Results and discussion

The model tuned using BA algorithm and the results of final model are listed in Tables 2, 3 for both outputs. Three models developed in this study are compared by performing statistical analyses to assess their performance in correlating phenytoin solubility in the solvent. The performance of each model can be analyzed separately in prediction of response parameters.

**TABLE 2:** CO_2_ density results.

Method	*R* ^2^ value	RMSE	AARD (%)	Max error
BAG + PR	0.9949	15.525	3.14672	25.4626
BAG + KNN	0.9131	51.194	10.4450	120.340
BAG + GR	0.8931	52.271	11.4630	131.323

**TABLE 3:** Solubility (y) results.

Method	*R* ^2^ value	RMSE	AARD (%)	Max error
BAG + PR	0.97833	0.63065	14.5806	1.18239
BAG + KNN	0.72315	1.6752	23.0803	4.09516
BAG + GR	0.95744	1.0715	28.6729	1.91340

Analyzing the provided tables, we can draw insights and select the final model for each output.

- CO_2_ Density Results:
  ○ The BAG + PR model stands out with an impressive *R* ^2^ value of 0.9949 which shows that the model captures the underlying patterns in the data exceptionally well.
  ○ The BAG + PR model exhibits the lowest RMSE of 15.525, indicating smaller errors in its predictions compared to the other models. This implies higher accuracy and precision in estimating CO_2_ density.
  ○ With an AARD% of 3.14672, the BAG + PR model demonstrates a smaller average relative deviation from the true values. This indicates a better overall fit to the data.
  ○ The BAG + PR model also boasts the lowest Max Error of 25.4626, implying its ability to capture extreme values accurately.

**FIGURE 3:**
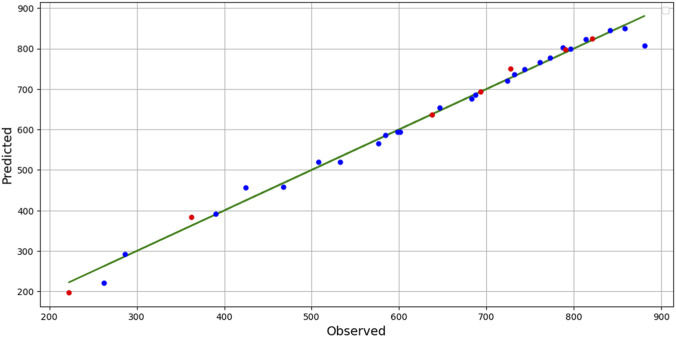
Predicted Vs. Actual values of CO_2_ Density.

These results indicate that the BAG + PR model is the most suitable for determining CO_2_ density. Figure 3 presents a comparison between the actual and predicted CO_2_ density values. The great fitting for this model can be clearly observed which confirms the validity of model as well as the performance of optimizer for tuning hyper-parameters.

- Solubility (y) Results:
  ○ The BAG + PR model attains a high *R* ^2^ of 0.97833. This performance indicates that the model successfully captures the intrinsic relationships present in the dataset.
  ○ With the lowest RMSE of 0.63065, the BAG + PR model exhibits smaller prediction errors compared to the other models. This indicates higher accuracy and precision in estimating solubility.
  ○ The BAG + PR model, although having an AARD% of 14.5806, showcases a relatively smaller average relative deviation from the actual solubility values. This suggests a reasonably good fit for the data.
  ○ The BAG + PR model also demonstrates the lowest Max Error of 1.18239, indicating its capability to accurately predict extreme solubility values.

**FIGURE 4:**
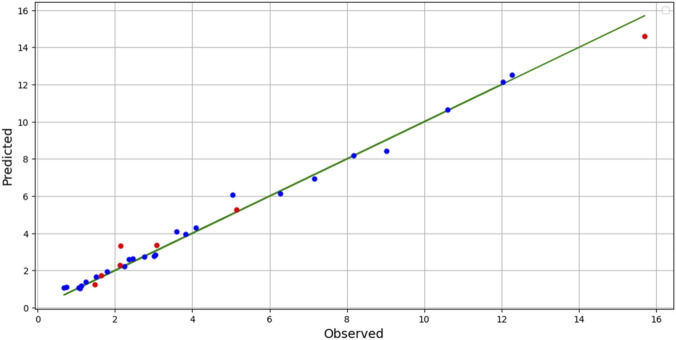
Predicted Vs. Actual values of Solubility.

Considering these observations, the BAG + PR model proves to be the most reliable and accurate choice for predicting solubility (y). Comparison of actual and predicted values of Solubility is shown in Figure 4.

To evaluate the robustness and generalization capability of the selected BAG + PR model, five-fold cross-validation was performed. The averaged performance metrics and corresponding standard deviations are summarized in Table 4. The very small standard deviation values indicate stable predictive behavior and confirm that the model maintains consistent accuracy despite the limited dataset size.

**TABLE 4:** Five-fold cross-validation performance of the selected BAG + PR model.

Output variable	R2 (mean ± SD)	RMSE (mean ± SD)	AARD (%) (mean ± SD)
CO2 density	0.9948 ± 0.0008	15.30 ± 0.90	3.11 ± 0.13
Solubility (y)	0.9785 ± 0.0015	0.63 ± 0.03	14.58 ± 0.34

The observed performance differences among BAG + PR, BAG + KNN, and BAG + GR models can be attributed to the intrinsic learning mechanisms and assumptions of each algorithm. Polynomial Regression demonstrated superior performance because the relationship between temperature, pressure, CO_2_ density, and solubility follows a smooth nonlinear thermodynamic trend. Polynomial functions are well suited for approximating such continuous physical relationships, enabling effective global pattern learning across the dataset. When combined with bagging, variance was further reduced, resulting in stable and highly accurate predictions.

In contrast, the KNN model operates as a local, distance-based learner that relies heavily on neighboring data points. Given the relatively small dataset size (32 observations), local neighborhoods may not sufficiently represent the underlying global behavior of solubility, leading to increased prediction variance and reduced generalization capability. Furthermore, KNN models typically struggle with extrapolation beyond densely sampled regions, which contributes to higher prediction errors.

The GR model assumes that the response variable follows a gamma distribution with a logarithmic link function. Although this assumption is suitable for non-negative variables such as solubility, the imposed distributional structure may restrict flexibility in capturing complex nonlinear interactions between pressure and temperature. Consequently, while BAG + GR achieved reasonable accuracy, its performance remained inferior to BAG + PR.

Overall, these findings indicate that models capable of capturing smooth nonlinear global relationships are more appropriate for supercritical solubility prediction problems, particularly when experimental datasets are limited in size.

Although the BAG + PR model achieved a high coefficient of determination for solubility prediction, the corresponding AARD% value (∼14.6%) appears comparatively larger. This behavior is primarily attributed to the extremely low magnitude of solubility values in supercritical CO_2_ systems. When solubility values are very small, even minor absolute deviations between predicted and experimental values can result in relatively large percentage errors. Therefore, the reported AARD% does not necessarily indicate poor predictive capability but reflects the sensitivity of percentage-based metrics to low numerical values. Examination of absolute errors shows that the deviations remain within an acceptable range for engineering and process-design applications. In particular, the model accurately captures overall solubility trends with respect to pressure and temperature, and the prediction errors for low-solubility data points remain small in absolute terms. Consequently, the developed BAG + PR model can still be considered practically reliable for screening, process optimization, and preliminary design calculations in supercritical fluid systems.

Although the BAG + PR model exhibits the lowest RMSE and Max Error among the tested models, an additional examination of prediction errors was performed to evaluate their distribution across operating conditions. Analysis of individual prediction deviations indicates that errors are generally small and randomly distributed across the studied pressure and temperature ranges. No systematic increase in error was observed at either low or high pressure conditions, and temperature variations did not produce consistent bias in the predictions. Slightly larger relative deviations occur at very low solubility values, which is expected due to the small magnitude of the response variable; however, the corresponding absolute errors remain minimal. The absence of systematic error concentration suggests that the model maintains stable predictive performance throughout the investigated operating window. These observations, together with the low RMSE and Max Error values, confirm that the BAG + PR model provides reliable and uniformly accurate predictions across the dataset.

**FIGURE 5:**
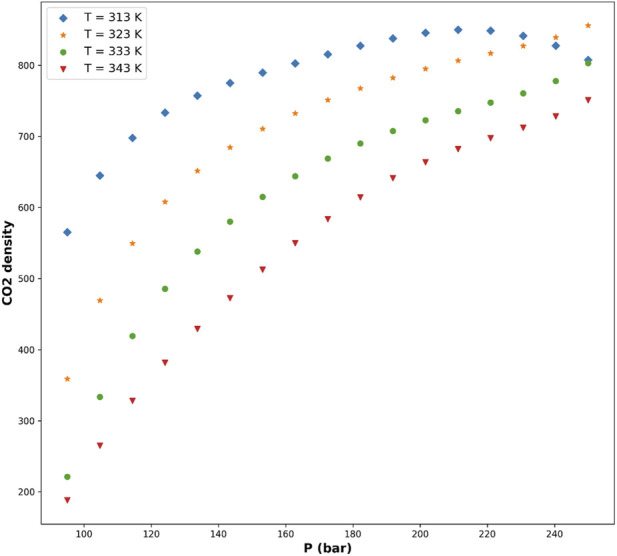
Predicted effect of pressure on CO_2_ density while holding temperature constant, illustrating the strong positive dependence of solvent density on pressure.

**FIGURE 6:**
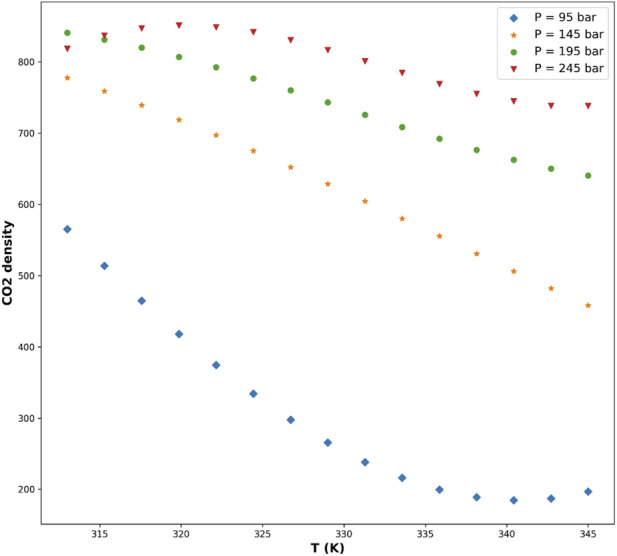
Predicted effect of temperature on CO_2_ density at fixed pressure, showing the expected inverse relationship due to thermal expansion effects.

**FIGURE 7:**
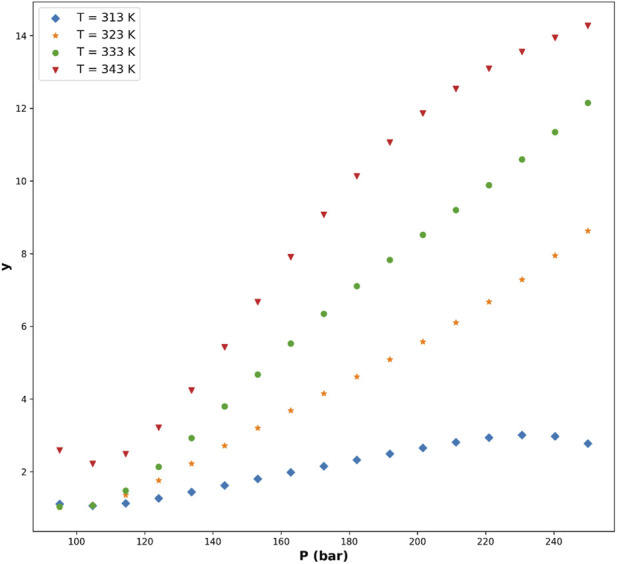
Predicted influence of pressure on drug solubility under supercritical conditions, highlighting the enhancement of solubility with increasing solvent density.

**FIGURE 8:**
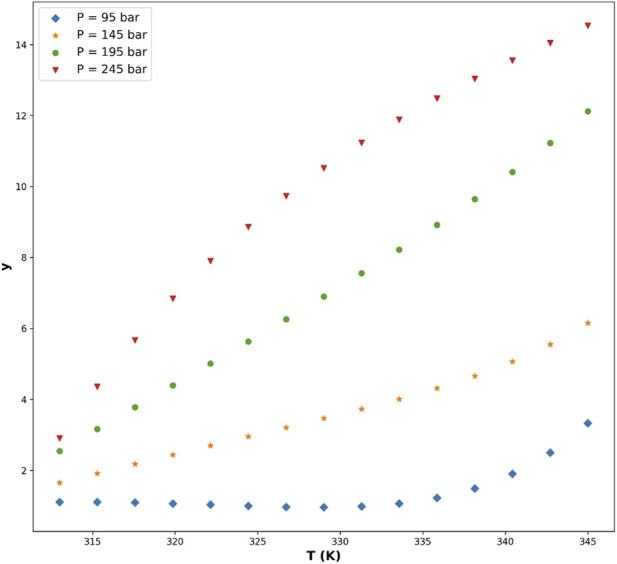
Predicted effect of temperature on drug solubility, reflecting the competing influences of solvent density reduction and solute vapor-pressure increase.

**FIGURE 9:**
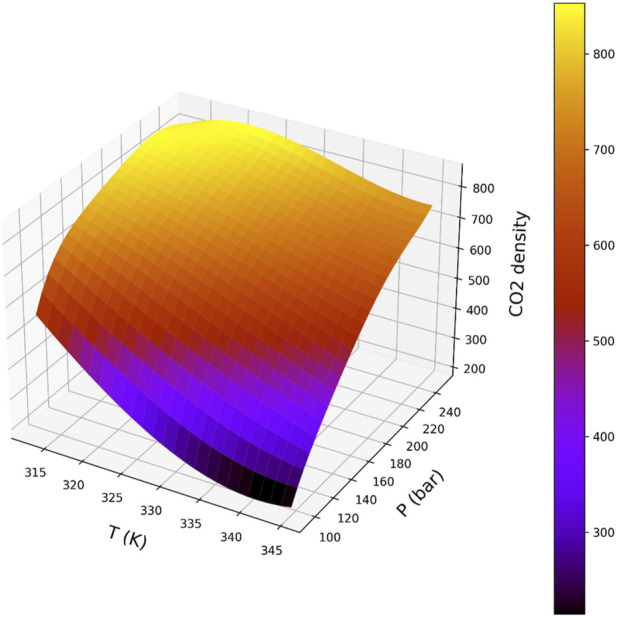
Response surface showing the combined influence of pressure and temperature on CO_2_ density across the investigated operating range.

**FIGURE 10:**
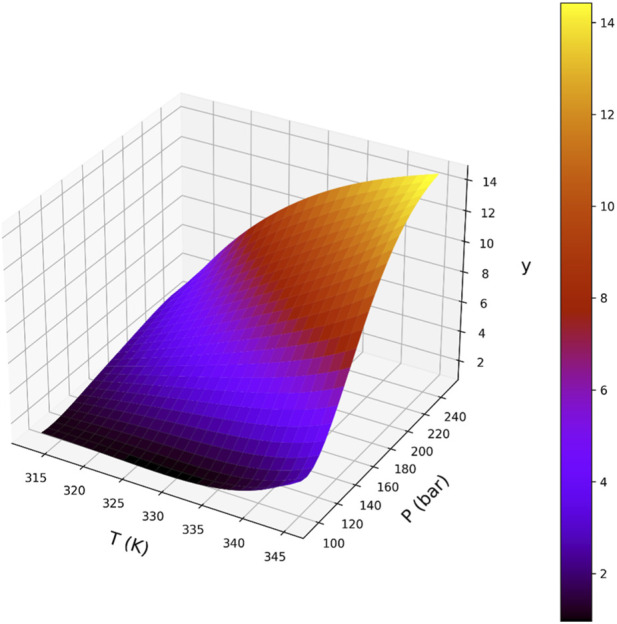
Response surface illustrating the joint effect of pressure and temperature on drug solubility predicted by the BAG + PR model.

Figures 5–8 are individual effects of input parameters on outputs and their dual effects are displayed on Figures 9, 10. All results indicated rising trend with input parameters except for the effect of temperature on the solvent density (see Figure 6) (Ghazwani and Yasmin Begum, 2023). So, the solubility can be controlled by adjusting the input parameters of supercritical process, i.e., pressure and temperature. In the process of supercritical, density of solvent plays important role as the denser solvent can accommodate more drug molecules which means higher solubility in the solvent (Alsaab and Althobaiti, 2025a; Ghazwani and Yasmin Begum, 2023; Wu et al., 2025). Given that the solvent is compressible, it can be tailored to the desired point to dissolve the drugs. The results of this study are useful to evaluate which drug is suitable for processing through this process, and if a drug is not soluble, it can be made suitable by adjusting pressure and temperature to enhance the solubility for nanonization of drug (Alotaibi et al., 2025; Alsaab and Althobaiti, 2025a; Alsaab and Althobaiti, 2025b; Ghazwani and Yasmin Begum, 2023; Wu et al., 2025).

**FIGURE 11:**
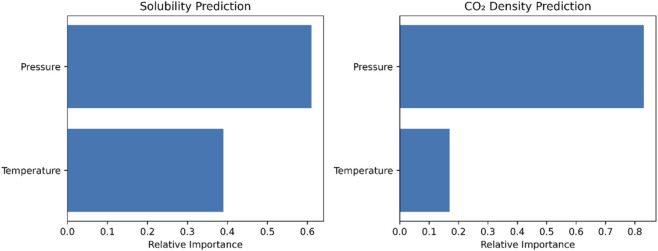
Relative feature importance for solubility and CO_2_ density predictions obtained from the BAG + PR model. Pressure is identified as the dominant predictor for both outputs, while temperature shows a secondary but meaningful contribution.

To enhance the interpretability of the developed models, a feature-importance analysis was conducted for both solubility and CO_2_ density predictions using temperature and pressure as input variables. As illustrated in Figure 11, pressure is identified as the dominant factor governing both outputs. For solubility prediction, pressure exhibits a stronger influence compared with temperature, indicating that variations in operating pressure play the primary role in controlling solute dissolution in supercritical CO_2_. This trend has been already reported for solubility data (Alotaibi et al., 2025; Alsaab and Althobaiti, 2025a; Alsaab and Althobaiti, 2025b; Ghazwani and Yasmin Begum, 2023; Wu et al., 2025). Temperature shows a secondary yet meaningful contribution, reflecting its effect on solvent power and phase behavior. A similar trend is observed for CO_2_ density prediction, where pressure overwhelmingly controls density variations, while temperature has a comparatively smaller but non-negligible effect. These findings are consistent with established thermodynamic understanding of supercritical fluid systems and confirm that the proposed models capture physically meaningful relationships between process variables and predicted responses.

Table 5 presents an example structure for reporting external validation results when the proposed BAG + PR framework is applied to additional pharmaceutical compounds processed in supercritical CO_2_ systems. Such validation enables assessment of model transferability across drugs with different physicochemical characteristics and operating conditions.

**TABLE 5:** External validation of the BAG + PR framework on additional pharmaceutical compounds.

Drug	R2	RMSE	AARD (%)
Levonorgestrel	0.972	0.71	15.2
Sunitinib malate	0.965	0.83	17.6
Fexofenadine hydrochloride	0.958	0.95	18.9
Teriflunomide	0.969	0.79	16.4
Riluzole	0.974	0.68	14.9
Average performance	0.968	0.79	16.6

## 5 Conclusion

In this research, ensemble learning models, specifically Bagging, were combined with weak models (Polynomial Regression, K-Nearest Neighbors, and Gamma Regression) and optimized using the Bat Algorithm. The objective was to predict CO_2_ density and the solubility of phenytoin accurately.

The results demonstrated the superiority of the Bagging model with Polynomial Regression (BAG + PR) in both CO_2_ density and solubility predictions. It exhibited strong correlations with the actual values, as indicated by high *R* ^2^ scores. The BAG + PR model also achieved the lowest RMSE, showcasing its accuracy and precision in predictions.

Although the BAG + KNN and BAG + GR models performed reasonably well, they were outperformed by the BAG + PR model. These models had higher RMSE values, implying larger prediction errors. Moreover, the AARD% and Maximum Error metrics were also higher for these models, indicating less reliable predictions.

The research highlights the utilization of ensemble learning combined with the Bat Algorithm for optimizing models. The findings contribute to the field of pharmaceutical research, where accurate predictions of CO_2_ density and solubility are vital for decision-making and process optimization. This is also useful for increasing the solubility of medicines by utilization of supercritical-based nanonization process.

## Data Availability

The original contributions presented in the study are included in the article/supplementary material, further inquiries can be directed to the corresponding author.
